# Self-Assembly Dynamics of Reconfigurable Colloidal Molecules

**DOI:** 10.1021/acsnano.1c09088

**Published:** 2022-01-26

**Authors:** Indrani Chakraborty, Daniel J. G. Pearce, Ruben W. Verweij, Sabine C. Matysik, Luca Giomi, Daniela J. Kraft

**Affiliations:** † Soft Matter Physics, Huygens-Kamerlingh Onnes Laboratory, Leiden Institute of Physics, PO Box 9504, 2300 RA Leiden, The Netherlands; § Department of Physics, Birla Institute of Technology and Science, Pilani - K K Birla Goa Campus, Zuarinagar, Goa 403726, India; ‡ Institute-Lorentz, Universiteit Leiden, PO Box 9506, 2300 RA Leiden, The Netherlands; ∥ Department of Mathematics, Massachusetts Institute of Technology, 182 Memorial Drive, Cambridge, Massachusetts 02142, United States; ∇ Department of Theoretical Physics, University of Geneva, Quai Ernest Ansermet 30, 1205 Geneva, Switzerland; ○ Yusuf Hamied Department of Chemistry, University of Cambridge, Lensfield Road, Cambridge CB2 1EW, United Kingdom

**Keywords:** structural flexibility, colloidal clusters, mobile DNA linkers, controlled valence, self-assembly

## Abstract

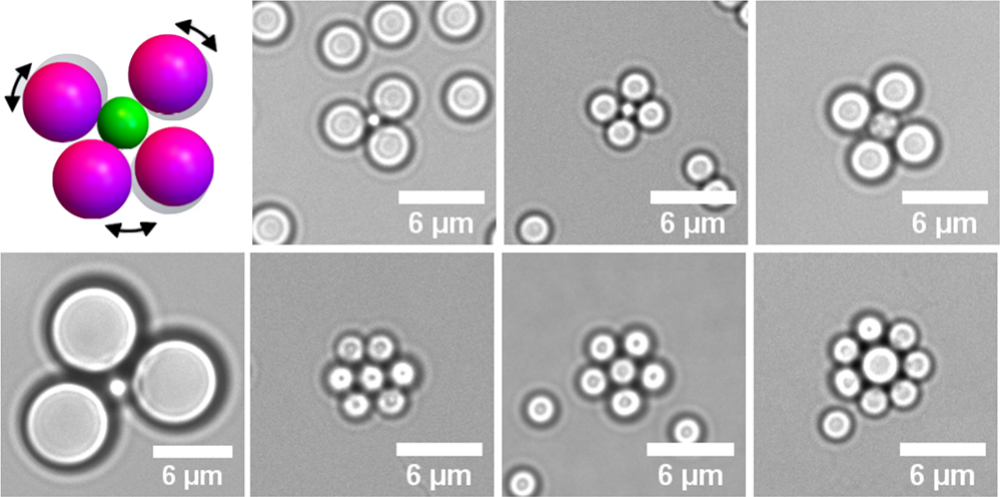

Colloidal molecules are designed to mimic their molecular analogues through their anisotropic shape and interactions. However, current experimental realizations are missing the structural flexibility present in real molecules thereby restricting their use as model systems. We overcome this limitation by assembling reconfigurable colloidal molecules from silica particles functionalized with mobile DNA linkers in high yields. We achieve this by steering the self-assembly pathway toward the formation of finite-sized clusters by employing high number ratios of particles functionalized with complementary DNA strands. The size ratio of the two species of particles provides control over the overall cluster size, *i.e.*, the number of bound particles *N*, as well as the degree of reconfigurability. The bond flexibility provided by the mobile linkers allows the successful assembly of colloidal clusters with the geometrically expected maximum number of bound particles and shape. We quantitatively examine the self-assembly dynamics of these flexible colloidal molecules by a combination of experiments, agent-based simulations, and an analytical model. Our “flexible colloidal molecules” are exciting building blocks for investigating and exploiting the self-assembly of complex hierarchical structures, photonic crystals, and colloidal metamaterials.

Colloidal particles are powerful model systems to study the assembly and phase behavior of atoms and molecules.^1^ Already, the simplest model, a colloidal sphere with isotropic interaction potential, has provided unparalleled insights into complex and fundamental processes such as crystallization,^2,3^ the glass transition,^4,5^ and melting/fracture.^6,7^ To capture the anisotropic interactions and shapes of more complex molecules, colloidal constructs made up of multiple “colloidal atoms” are being developed and further functionalized with site-specific interactions by various approaches.^8^ In analogy with their molecular cousins, they are called “colloidal molecules”^9−11^ and are expected to revolutionize the field of material science due to their tunable complexity and additional control over the assembly process. However, with a few exceptions, their syntheses consist of many step processes.^12−16^ The simpler techniques often produce a wide range of colloidal molecules that require time-consuming separation protocols before they can be employed in experiments,^17,18^ and the low yield for certain cluster sizes limits their usability in experiments.^18,19^ Furthermore, despite their shape and interaction analogy, all current realizations of colloidal molecules consist of rigid objects that fail to mimic the structural flexibility present in real molecules. Yet, structural reconfigurability is essentially the property that has been predicted to strongly affect the assembly and phase behavior of colloidal molecules, and any rigid system will have limited use as a model system.^20^

Recent theoretical studies on colloidal molecules with such structural reconfigurability have found a variety of unusual behaviors, such as the appearance of crystalline lattices^21,22^ and other ordered structures, that are kinetically inaccessible for rigid colloidal molecules.^23^ For instance, hierarchically assembled flexible colloidal molecules have been numerically shown to give rise to thermodynamically stable liquid–liquid phase separation.^22^ This long sought-after phase transition may explain the origin of the anomalies of liquid water but is difficult to probe experimentally. With increasing bond flexibility, the crystalline phase has been furthermore predicted to be replaced by a previously unexpected state of matter, the fully bonded disordered network state.^23^ To date, none of these predictions have been experimentally validated because of the rigidity of the current experimental realizations of colloidal molecules.

Here, we exploit colloidal elements that provide flexibility between linked particles, so-called colloidal joints,^24,25^ to assemble different types of flexible colloidal molecules with high fidelity and yield. We achieve this by choosing a high number ratio of two sets of spheres functionalized with complementary, surface-mobile DNA strands and assemble them into small clusters, where one sphere type surrounds the spheres of the other type.^26−28^ Depending on their size ratio and thus packing density of the spheres on the outside, colloidal molecules with different degrees of flexibility and cluster sizes, *i.e.*, the number of bound spheres, can be realized. We investigate the growth dynamics of colloidal molecules with different maximum numbers of bound spheres using experiments and agent-based simulations and describe it quantitatively by a theoretical model that considers the availability of bonding space on the surface of the central particle. The high yield combined with a tunable flexibility and controlled size of our flexible colloidal molecules makes them not only an excellent model system for studying the phase behavior of molecules but also exciting building blocks for creating reconfigurable materials or bits in wet computing.^28−30^

## Results and Discussion

### Assembly Strategy

**Figure 1:**
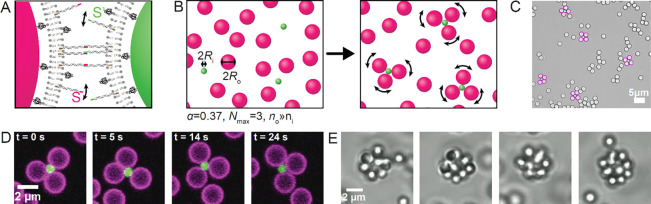
Self-assembly of reconfigurable colloidal molecules. (A) Schematic diagram of the self-assembly process. Combining two types of colloidal spheres (*S* and *S′*) functionalized with complementary DNA linkers (indicated in magenta and green) at high number ratios leads to the formation of finite size clusters. The flexible bond is formed through linkages between surface mobile DNA linkers anchored into a lipid bilayer on the particle surface. (B) Schematic showing colloidal spheres with a radius ratio (α) of 0.37 and maximum number of bound particles (*N*_max_) of 3, assembling into colloidal molecules when the density of the outer spheres, *n*_o_, is much greater than that of the inner spheres, *n*_i_. The surface mobility of the DNA linkers imparts reconfigurability to the bonded particles. (C) Bright-field microscopy image of the self-assembled colloidal molecules for α = 0.67. Clusters with *N*_max_ = 4 are highlighted by colored outlines. Scalebar is 5 μm. (D) Time-lapse confocal images of a quasi-2D reconfigurable colloidal molecule composed of 2.06 ± 0.05 μm silica particles (magenta) surrounding a central 1.15 ± 0.05 μm silica particle (green). See Movie S1 for corresponding video. (E) Time-lapse bright field images from Movie S2 of a 3D colloidal molecule composed of 1.15 ± 0.05 μm polystyrene particles encompassing a central 2.06 ± 0.05 μm silica particle. The lower density of the polystyrene particles enables the polystyrene particles to diffuse on the curved surface of the central sphere.

To create flexible colloidal molecules, we follow a strategy based on assembling spherical particles onto the surface of a central sphere.^26,31−33^ The size ratio α = *R*_i_/*R*_o_ of the two spheres determines the cluster geometry by simple packing arguments, where *R*_i_ and *R*_o_ are the radius of the inner and the outer sphere, respectively.^26,34^ To steer the assembly pathway toward finite size clusters, the outer particle species is used in excess of the particle species intended to form the core of the cluster; see Figure 1A,B. Until now, the experimental realization of this straightforward idea, however, has struggled with what has been termed the “random parking” problem: when two bonding particles cannot rearrange, which is the case for interactions based on surface-bound DNA linkers^31^ and charge,^31,33,35,36^ the optimal packing of the outer particles cannot be achieved.^31^ This precluded the formation of the geometrically predicted clusters and led to the formation of clusters with nonuniform shapes and sizes. Even the flexible bonds between lock-and-key shaped particles mediated by depletion interactions could not achieve the assembly of only one type of cluster due to kinetic barriers.^37^ The assembly of a single flexible tetrahedral cluster by holographic optical tweezers was demonstrated as a proof of principle.^28^ However, while this method is very useful to study the dynamics of individual colloidal molecules with internal degrees of freedom,^20,28,38^ the time-consuming particle-by-particle addition is not a viable approach for the production of more than a few individual flexible colloidal molecules.

To resolve the “random parking” problem and overcome kinetic barriers, we use colloidal joints as the central particle of the cluster.^24,25^ Colloidal joints are particles that provide the same structural flexibility between linked colloids as their macroscopic analogues by exploiting DNA linkers that are mobile on the particle surface.^39^ Experimentally, we realize colloidal joints by coating silica particles of diameters 1.15 ± 0.05, 2.06 ± 0.05, 3.0 ± 0.25, and 7.0 ± 0.3 μm with a closed lipid bilayer into which DNA linker strands with double cholesterol moieties are anchored (Figure 1A). At room temperature, the bilayer is in the liquid phase (the transition temperature *T*_m_ of DOPC is −17 °C),^40^ allowing free diffusion of the cholesterol-anchored DNA linkers on the particle surface (Figure 1A). See the Methods for the experimental details. Colloidal particles connected to DNA linkers that are mobile on the surface inherit their ability to freely diffuse over the surface of the colloidal joint particle (Figure 1B). This surface mobility enables us to obtain clusters with maximum packing (Figure 1C) as the outer particles can *rearrange* after binding and thereby provide access to the core particle surface for additional oncoming particles until saturation and hence the geometrically expected maximum number of outer particles has been reached. At the same time, it is the crucial element to create internal degrees of freedom in the colloidal molecules. By choosing DNA linkers with 11 base pair long single stranded sticky ends, we ensure essentially irreversible binding between particles even at high packing densities and overcome any kinetic barriers.

By combining flexible joints functionalized with DNA linker strands *S′* (represented by green particles in Figure 1A–C) with an excess of particles functionalized with the complementary strand *S* (represented by magenta particles in Figure 1A–C), we obtain finite-sized colloidal clusters with full flexibility of the attached outer spheres or reconfigurable colloidal molecules (see the Methods section). We can experimentally realize quasi-2D (Figure 1D) or 3D (Figure 1E) flexible colloidal molecules by simply selecting the material of the outer spheres and its associated gravitational height in water. When we use solid silica spheres with surface-mobile DNA linkers *S* and *S′* as the outer and inner particles of the cluster, the experiment is confined to quasi-2D; see Figure 1D and Movie S1. The employment of lower-density polystyrene beads with surface-bound DNA linkers *S* relieves this constraint for the outer particles and leads to flexible colloidal molecules where the outer particles are free to move in three dimensions, as shown in Figure 1E and Movie S2. Since we chose solid silica spheres for the inner particles, their motion is still confined to quasi-2D. This choice was necessary because flexibility requires a high-quality lipid bilayer that can only be achieved on silica surfaces. In the future, colloidal molecules that can fully explore three dimensions might be realized by using particles that only feature a silica surface and are either hollow or filled with a material whose density is similar to that of the solvent. For simplicity, we restrict ourselves to the quasi-2D case in the following experiments and discussions.

### Reconfigurable Colloidal Molecules with Size Tunable by Geometry

**Figure 2:**
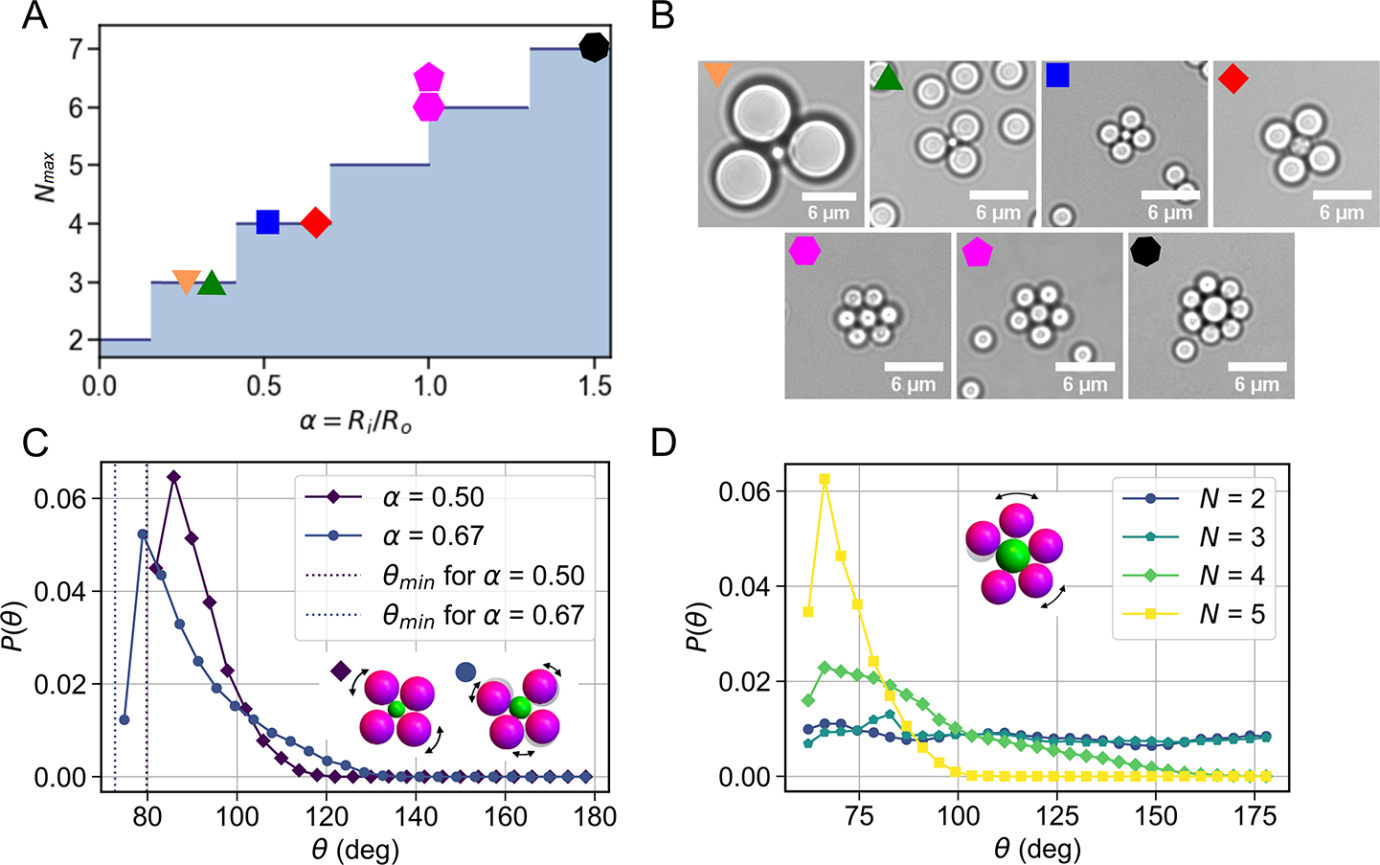
Reconfigurable colloidal molecules of different size ratios α and corresponding valences *N*_max_ of the central particle. (A) Geometrically expected *N*_max_ for different size ratios α and (B) the corresponding bright field images of the obtained colloidal molecules at select ratios: orange ▼, α = 0.28, 2*R*_i_ = 2.06 ± 0.05 μm, 2*R*_o_ = 7.0 ± 0.3 μm; green ▲, α = 0.33, 2*R*_i_ = 1.15 ± 0.05 μm, 2*R*_o_ = 3.0 ± 0.25 μm; blue ■, α = 0.5, 2*R*_i_ = 1.15 ± 0.05 μm, 2*R*_o_ = 2.06 ± 0.05 μm; red ◆, α = 0.67, 2*R*_i_ = 2.06 ± 0.05 μm, 2*R*_o_ = 3.0 ± 0.25 μm; pink ⬣ and ⬟, α = 1, 2*R*_i_ = 2.06 ± 0.05 μm, 2*R*_o_ = 2.06 ± 0.05 μm; black heptagon, α = 1.5, 2*R*_i_ = 3.0 ± 0.25 μm, 2*R*_o_ = 2.06 ± 0.05 μm. Here, α = *R*_i_/*R*_o_ where *R*_i_ and *R*_o_ are the radii of the inner (core) and outer particles, respectively. For α = 1, the majority of the colloidal molecules had *N* = 5 outer particles, and occasionally, *N* = 6 was observed. (C) Experimental probability distribution *P*(θ) of the angle θ between any two adjacent outer spheres in a colloidal molecule for α = 0.5 and α = 0.67 shows that the angular motion range of the outer spheres for a given maximum valence (*N*_max_ = 4 here) is tunable through the size ratio and is larger for higher values of α. The inset shows the schematics of the two resulting clusters (not to scale to illustrate different available space). (D) The angular motion range decreases as *N* increases as can be seen from *P*(θ). The data shown stems from experiments for α = 1 (*N*_max_ = 6) for *N* = 2, 3, 4, and 5.

The size and thus number of outer spheres of our reconfigurable colloidal molecules can be straightforwardly controlled through the choice of the size ratio α = *R*_i_/*R*_o_. A central particle of a colloidal molecule with *N* outer spheres is considered to have valence *N*, which is equivalent to the number of outer spheres or the cluster size. For a closed packed combination of hard spheres in 2D, the maximum valence *N*_max_ of the central particle in the corresponding 2D cluster is given by^26,41^
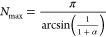
1purely from geometrical considerations. Also, see the Supporting Information for additional considerations about the impact of different heights of the constituent particles. In a physical system, *N*_max_ can only have integer values, whereas the fractional part of *N*_max_ accounts for gaps in between the spheres. Taking the integer part of *N*_max_ yields a stair curve^26^ with increasing α; see Figure 2A. We demonstrate the assembly of flexible colloidal molecules with *N*_max_ = 3 to *N*_max_ = 7 by making various combinations of differently sized spheres as outer or inner particles of the colloidal molecules. The results are displayed in Figure 2B with symbols denoting the respective size ratios in Figure 2A. For example, for a radius ratio of α = 0.29, we use an excess of 7.0 ± 0.3 μm spheres in combination with few 2.06 ± 0.05 μm spheres to obtain clusters with *N*_max_ = 3; see Figure 2A,B. In contrast to previous attempts at assembling clusters through this approach, we here find that the experimentally obtained *N*_max_ perfectly agrees with the values expected on the basis of the size ratio of the constituent spheres. This is the direct consequence of the reconfigurability of the system, which solves the random parking problem and allows the assembly of many different types of flexible colloidal molecules with the expected maximum valences.

Interestingly, each cluster size *N*_max_ can be formed within a range of size ratios α. This is represented by the finite width of each step for every integer value of *N*_max_ (Figure 2A). At the lower end of the size ratio for any given *N*_max_, the outer spheres are closely packed, while at the higher end there is sufficient space to almost fit an additional particle and thus more area is available to move. Therefore, the choice of the size ratio α also provides a handle to control the range of angular motion of the colloidal molecules and hence their flexibility. We note that the term “flexibility” has been used to describe both the angular motion range^23^ and the angular speed of the outer particles.^20,24^ Angular motion range and angular speed may both contribute to the intuitive meaning of flexibility, and we hence use it in this way here. To show that the size ratio of the two sphere types enables a tunable motion range, we assemble colloidal molecules with *N*_max_ = 4 from spheres with size ratio α = 0.5 and α = 0.67. Visual inspection of the resulting colloidal molecules confirms that the outer spheres of α = 0.67 colloidal molecules have more space to move on the surface of the central particle than they do for α = 0.5 colloidal molecules; see Movie S3. We quantify the angular motion range of the two types of *N*_max_ = 4 colloidal molecules by measuring the probability distribution *P*(θ) of the angle θ between any two outer spheres and the central particle (Figure 2C). Colloidal molecules with α = 0.67 show a wider spread in the angular distribution indicating a higher degree of flexibility, while the angular motion of the spheres in the more closely packed α = 0.5 cluster is more constrained. In addition, the minimum angle that is geometrically possible as well as the most probable angle shifts toward larger angles with decreasing size ratio due to the larger outer particles, pointing yet again toward a stronger confinement of the outer spheres for smaller size ratios.

The motion range also decreases with increasing valence *N* of the inner particle during the assembly process. To investigate this quantitatively, we plot *P*(θ) for α = 1 (*N*_max_ = 6) from *N* = 2 to *N* = 5 (Figure 2D). While flexible colloidal molecules with *N* = 2 and *N* = 3 show a flat angular distribution, a peak appears for *N* = 4 that becomes pronounced at *N* = 5. This shift in the angular distribution is again connected to the increasingly constrained motion of the outer spheres. We note that this confinement may also affect the speed with which the outer spheres can move over the surface of the central particle.^20^ Because the distances between the outer particles have an effect on their angular speed through hydrodynamic interactions, the flexibility is expected to decrease as a function of increasing valence *N* due to both a smaller angular range and a lower angular speed. Control over the flexibility is particularly exciting in view of the predicted impact on the phase diagram of limited valence particles.^23^ Tuning of the size ratio of the constituent particles thus enables us to obtain a wide variety of colloidal molecules with both controlled size *N*_max_ and tunable flexibility.

### Dynamics of Colloidal Molecule Growth

To reach high yields of colloidal molecules with maximum valence of the central particle, we need to get a better understanding of the dynamics of their self-assembly process. We examine this process by a combination of experiments and agent-based simulations. For the experiments, we chose (a) 1.15 ± 0.05 μm silica spheres as the core particle and 2.06 ± 0.05 μm silica spheres as the outer particles and (b) 2.06 ± 0.05 μm silica spheres as the core particle and 3.0 ± 0.25 μm silica spheres as the outer particles, both cases corresponding to an expected maximum valence *N*_max_ = 4. The particles were mixed in a number ratio of 1:5 and 1:20 to obtain good statistics, and the growth of the colloidal molecules was observed for 1.2 days. At this number ratio, a few colloidal polymers^24,38,42^ and other composite structures were produced besides the colloidal molecules, which were excluded from the total count to enable a comparison with the simulations. The population growth at different *N*’s including and up to the expected maximum *N*_max_ = 4 were recorded at fixed time intervals.

**Figure 3:**
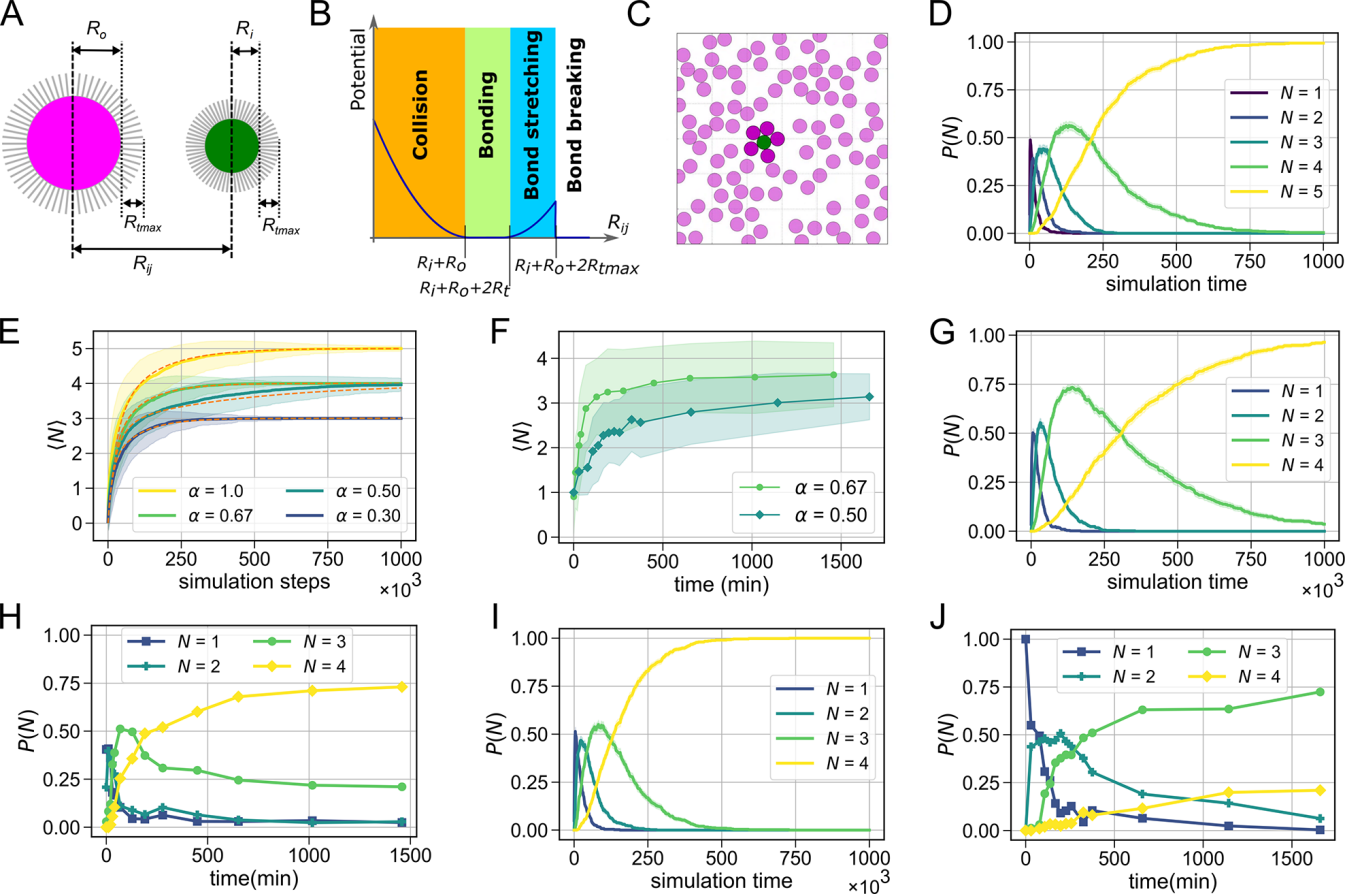
Comparison between experiments and agent-based simulations of the self-assembly dynamics of a colloidal molecule. (A) Schematic diagram of the simulation setup. Two disks with radii *R*_i_ and *R*_o_ each have linkers of maximum length *R*_tmax_ on their surface and interact with (B) a distance dependent interaction potential, which is shown for a value of *R*_i_ + *R*_o_ = 4*R*_t_ = 2*R*_tmax_. The interaction force is repulsive when *R*_ij_ < *R*_i_ + *R*_o_; it is zero in the range of *R*_i_ + *R*_o_ < *R*_ij_ < *R*_i_ + *R*_o_ + 2*R*_t_ and attractive when *R*_i_ + *R*_o_ + 2*R*_t_ < *R*_ij_ < *R*_i_ + *R*_o_ + 2*R*_tmax_ until bond breaking occurs for *R*_ij_ > *R*_i_ + *R*_o_ + 2*R*_tmax_. (C) Representative still from the simulation of a reconfigurable colloidal molecule for α = 1.0. The core particle (green) is surrounded by an excess of complementary particles (magenta). (D) The simulated probability *P*(*N*) of finding a cluster with valence *N* as a function of time for α = 1 shows that the assembly saturates at *N* = *N*_max_ – 1. (E) Simulated (solid line) and calculated (orange dashed line) ensemble averaged numbers of bound particles ⟨*N*⟩ for α = 0.3, α = 0.5, α = 0.67, and α = 1.0 as a function of simulation steps show excellent agreement. (F) Experimentally obtained ensemble averaged ⟨*N*⟩ for α = 0.5 and α = 0.67, respectively, as a function of time. (G) Simulated and (H) experimentally measured *P*(*N*) for α = 0.67. (I) Simulated and (J) experimentally measured *P*(*N*) as a function of time for α = 0.5.

We complemented these experiments with agent-based simulations to examine the self-assembly process for a range of values of α. The colloidal spheres were approximated as two-dimensional soft disks with radii *R*_i_ and *R*_o_, which represent the central and outer particles, respectively; hence, *R*_i_ = α*R*_o_ (Figure 3A). Each disk undergoes an uncorrelated random walk with a constant step length *T*. When two simulated disks come into contact, the distance between the centers of the two disks is less than the sum of their radii, *R*_ij_ < *R*_i_ + *R*_o_. This results in a small overlap *d*, which we use to define a repulsive force *F*_R_ = *K*_disk_*d* directed away from each other (Figure 3B), where *K*_disk_ is a constant. Each disk is assigned a certain type of DNA tether, *S* (or *S′*), with a rest length *R*_t_ and a maximum length *R*_tmax_. If the distance separating two disks with complementary DNA tethers is less than *R*_i_ + *R*_o_ + 2*R*_t_, then a bond is formed between these disks (Figure 3B). This bond behaves like a linear spring when *R*_ij_ > *R*_i_ + *R*_o_ + 2*R*_t_; *i.e.*, there is an attractive force *F*_A_ = max [*K*_tether_ × (*R*_ij_ – *R*_i_– *R*_o_– 2*R*_t_), 0] with *K*_tether_ being a constant representing the spring stiffness. This force is merely an attractive force having no torsional effect on the disk and replicates the mobility of the DNA linkers used in experiments. If the distance between two bound disks exceeds *R*_i_ + *R*_o_ + 2*R*_tmax_, the bond between them is broken, implying the bonds have a finite strength. The resulting energy landscape for bonded disks contains a wide minimum representing the range of separation between two bonded disks at which they apply no forces to each other. This is essentially some additional “wiggle room” provided by the nonzero length of the DNA linker. All simulations were performed with a single particle of radius *R*_i_ surrounded by a bath of 99 particles of radius *R*_o_ (Figure 3C and Movie S4). Simulations were initialized by placing particles at random, nonoverlapping positions in a periodic box of length *L*. We scale all lengths by *R*_o_ and control *R*_i_ by adjusting α and fix *R*_t_ = 0.02. We set  giving a constant packing fraction of π/9. The remaining parameters are set to *K*_disk_ = *K*_tether_ = 1, *R*_tmax_ = 10*R*_t_, and *T* = 0.01. Reported values were obtained by averaging over 500 simulations all with the number ratio of 1:99.

We expect that the available space on the surface of the central particle not only influences the flexibility but also directly influences the speed with which the clusters reach full saturation. Intuitively, the assembly should proceed faster if more space is available on the central particle for binding another outer particle. This is clearly observed in the simulated probability *P*(*N*) for all colloidal molecules that have bound *N* particles for α = 1.0 (Figure 3D). As we go from *N* = 1 to *N* = 5, the peak becomes broader and broader, indicating a slowdown of the growth process. We quantitatively investigate this by comparing the evolution of the average number of spheres bound to the central particle, ⟨*N*⟩, for four values of α, namely, α = 0.30, α = 0.50, α = 0.67, and α = 1.0 in the simulations (Figure 3E). We find that ⟨*N*⟩ increases monotonically with time toward the maximum allowed number. Initially, growth is fast and then slows down while saturating at a maximum valence. The same behavior of ⟨*N*⟩ is also obtained in our experiments for α = 0.5 and α = 0.67; see Figure 3F. An increasing size ratio for the same value of *N*_max_ indeed leads to a speed up of the assembly process; see α = 0.5 and α = 0.67 for *N*_max_ = 4. The flexibility and assembly speed and, ultimately, the yield, are thus directly related to the size ratio of the two spheres.

The maximum valence of the core particle shown in Figure 3E agrees with the geometrically predicted one for α = 0.30, α = 0.50, and α = 0.67, but surprisingly, only reaches *N* = 5 instead of the expected *N*_max_ = 6 for α = 1.0. We observed a similar behavior in the experiments, where the majority of colloidal molecules for α = 1.0 features at maximum *N* = 5 instead of 6. For this reason, we show examples of both *N* = 5 and *N*_max_ = 6 clusters for α = 1.0 in Figure 2B. A careful look at the bright field image in Figure 2B provides insight as to why colloidal molecules in experiments occasionally reach *N*_max_ = 6: the outer spheres have slightly different scattering patterns, indicating a variation in height from the surface. While simulations are restricted to 2D, the quasi-2D nature of the experiments allows out-of-plane diffusion. This together with a (small) size polydispersity of the colloidal particle creates additional space to access and bind to the core particles, albeit with low probability. The majority of clusters, however, saturates at a valence *N* = *N*_max_ – 1 for α = 1.0. This behavior can be understood from an entropic argument: to achieve *N*_max_, the outer particles need to be tightly packed around the core particle in a single state, leaving no space for internal motion. This state is entropically unfavorable compared to the large number of nonclose-packed arrangements that are possible due to the internal flexibility; also, see the distribution of angles between outer particles shown in Figure 2C. This submaximum saturation behavior occurs for all maximum sizes (*N*_max_) at the smallest size ratio at which they still can be assembled or, in other words, just after the transition from one maximum valence to the next higher one.

To further compare experiments and simulations, we looked at the assembly dynamics for *N*_max_ = 4 and α = 0.5 and α = 0.67. For this, we plot the probability *P*(*N*) for all colloidal molecules that have bound *N* particles for both simulations (Figure 3G,I) and experiments (Figure 3H,J). The binding of the first particle occurs very fast, and all core particles quickly have one particle bound. This still leaves ample surface available for binding a second particle and, indeed, leading to a sharp peak of *P*(1) and a quick increase of *P*(2). The addition of the third particle shows a slight slowdown but still occurs at a rate comparable with that characterizing the binding of the first two particles, consistent with the almost θ-independent angular motion range shown in Figure 2D. With the significantly reduced available space on the surface and the surface mobility of the bound particles, which hinder access to this surface further, the binding of the final particle now clearly slows down. In the experiments for α = 0.67, 71% of the core particles attain a valence *N* = 4 within 1000 min (Figure 3H), whereas only 17.4% attain *N* = 4 for α = 0.5 within 1000 min (Figure 3J). Note that the assembly is faster for the α = 0.67 case despite the slow diffusion of the larger outer particles (3 μm diameter) as compared to the case of α = 0.5 (outer particles having a 2 μm diameter). This clearly indicates that, once the core particles are surrounded by an excess of outer particles, the dominating factor that controls the speed of the assembly is the available space on the core particle surface, which in turn is decided by the size ratio α.

Despite the gradual slowdown of the assembly process, a reasonably good yield of flexible colloidal molecules with a maximum number of outer particles could be obtained simply by introducing reconfigurability to the system. Nonreconfigurable clusters typically yield distributions of cluster sizes due to the random distribution of the outer spheres, which can only be circumvented for *N*_max_ = 2 and *N*_max_ = 4 by choosing a specific α.^31^ Here, in this work, the reconfigurability in principle enables optimization for any size ratio and maximum valence. However, the self-assembly of colloidal molecules with a given *N*_max_ progresses faster for larger size ratios: for α = 0.5, we obtained 21% of *N*_max_ = 4 colloidal molecules in a time interval of 1.2 days while for α = 0.67 it was 73% in a time interval of 1.0 day; see Figure 3J,H, respectively. The yields could be further improved by using inert DNA or PEG linkers^25^ on the colloidal joints to passivate any remnant nonspecific interactions in the system and by a thorough passivation of the bottom glass surface of the sample chamber so as to let the self-assembly process continue unhindered over days. Altogether, our simple model captures the behavior of the experimental system well.

### Analytic Model for the Assembly Dynamics of Flexible Colloidal Molecules

We now attempt to turn to our hypothesis that the self-assembly dynamics are governed by the available surface area on the core particle into an analytical model. For this, we assume that the formation of the colloidal molecules is a diffusion-limited process of successive collisions between the central *S′* particle (green) with incident *S* particles (magenta). Incident particles have probability *p*_*N*_(α) of forming a bond with a cluster already containing *N* – 1 particles and with size ratio α. Using an independent Poisson process to model the addition of particles, the probability of having added the *N*^th^ particle to a cluster currently containing *N* – 1 particles in a time interval δ*t* can be described by the cumulative density function (CDF)
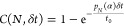
2

**Figure 4:**
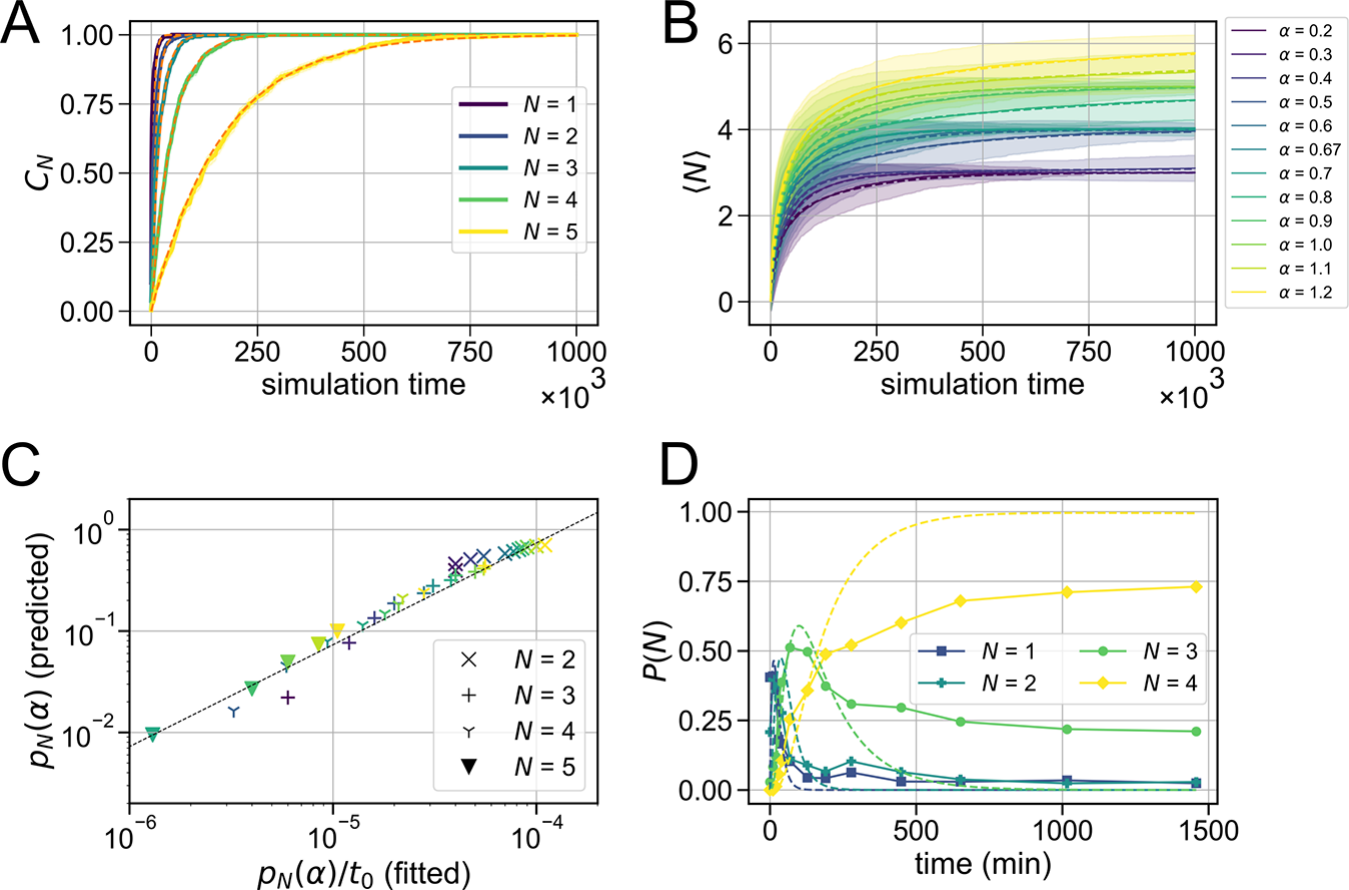
Analytical model of the assembly of a reconfigurable colloidal molecule. (A) Cumulative probability (simulated) of adding the *N*^th^ particle to a cluster for α = 1.0 (solid lines), plotted alongside eq 2 (orange dashed lines), in which we have used regression to fit for the value of *p*_*N*_(α)/*t*_0_. (B) Average growth (simulated) of the ⟨*N*⟩ of a cluster for a set of values of α ranging from 0.2 to 1.2 (solid lines) along with analytically obtained curves obtained using the values of *p*_*N*_(α)/*t*_0_ found by regression (dashed lines); see the Supporting Information. (C) Comparison of *p*_*N*_(α)/*t*_0_ obtained by regression to *p*_*N*_(α) predicted by the entropic theory. The dashed line shows a linear relationship with *t*_0_ = 7.5 × 10^3^. (D) Fit of the analytical model (dashed line) to the experimentally obtained *P*(*N*) vs time plots (solid lines) for α = 0.67. While the fit is good at shorter time intervals, it diverges at longer times.

Figure 4A shows the cumulative probability (simulated) of adding the *N*^th^ particle to a cluster for α = 1.0 (solid lines), plotted alongside eq 2 (orange dashed line), in which we have used regression to fit for the value of *p*_*N*_(α)/*t*_0_. By combining the CDFs (eq 2) for subsequent cluster sizes with the corresponding probability density functions (PDFs), *i.e.*, their time derivatives, we obtain an analytic expression for the expected number of bound particles as a function of time. Using the values of *p*_*N*_(α)/*t*_0_ found by regression, this can be used to predict the average growth of a cluster for a range of values of α, Figure 4B (see the Supporting Information and Figure S1 for details).

Since the system is isotropic, we can assume that an incident particle can arrive from any direction and, therefore, also estimate the values of *p*_*N*_(α) as the fraction of configurations that allow for the addition of an *N*^th^ particle to a cluster with *N* – 1 bound particles. To calculate this, we describe a cluster by the angles between the bound particles. Without the loss of generality, we fix the angular position of the first bound particle to be θ_1_ = −φ, where φ is the minimum angle allowed between two particles shown in Figure S2 and given by cos(φ) = 1 – 2*R*_0_^2^(*R*_i_ + *R*_o_ + 2*R*_t_)^−2^. The angular positions of the remaining particles are then labeled sequentially in a clockwise manner. The available angular space for a second particle is then simply 2π – 2φ and thus *p*_2_(α) = 1– φ/π. The addition of a third particle will now depend on the position of the second particle. If θ_2_ ≈ 0, there will be available space for the addition of a third particle in the region θ_3_ ∈ [θ_2_ + φ, 2π – 2φ]; however, if θ_2_ ≈ 2π – 2φ, the third particle will only have space within the region θ_3_ ∈ [0, θ_2_ – φ]. To account for all possible configurations, we must integrate both scenarios over all possible positions of θ_2_ to find *p*_3_(α) = [3φ – 2π]^2^/[2π(2π – 2φ)] (see the Supporting Information and Figure S3). A similar process can be followed for every subsequent particle that is added, detailed in the Supporting Information. When comparing the estimates of *p*_*N*_(α)/*t*_0_ obtained *via* regression to those predicted by this entropic theory, we find a linear relationship; see Figure 4C. By performing a linear fit in Figure 4C, we can estimate the simulation value of *t*_0_ = 7.5 × 10^3^. Furthermore, we can show that the characteristic time, *t*_0_, scales linearly with the particle radius, *R*_o_, inversely with the squared packing fraction, Φ^2^, and inversely with the squared step length, *T*^2^, in the simulation, 1/*t*_0_ ∼ *T*^2^Φ^2^/*R*_0_ (see the Supporting Information). We plot the prediction of the aforementioned model (orange dashed lines) alongside the simulation results (solid lines) with the single fitting parameter *t*_0_ = 7.5 × 10^3^ in Figure 3E.

We can now match our analytic model to the experimental results for α = 0.67. To do so, we experimentally measured the probabilities *P*(*N*) for a single flexible colloidal molecule to have *exactly N* outer particles as a function of time. We subsequently made least-squares fits to this data using the above expressions for *p*_2_, *p*_3_, and *p*_4_. As shown in Figure 4D, we found good overall agreement for α = 0.67 at shorter time intervals. The deviation at longer time intervals might stem from a slowdown in the assembly process due to nonspecific interactions between the particle surface and the bottom glass surface of the sample chamber or due to degeneration of the lipid bilayer over several hours. It has been recently shown that, by inserting lipopolymers of required molecular weights or double stranded inert DNA linkers in the lipid bilayers, this problem of nonspecific interactions over extended time periods can be solved to a large extent.^25^

The assembly dynamics of flexible colloidal molecules can be summarized as being fast initially and then gradually slowing down with an increasing number of bound particles. This behavior can be explained by the fact that, since there are no long-range attractive forces present in the system, an oncoming particle has to come in close proximity to the central particle and stay there for a sufficiently long time so as to form a linkage. Therefore, a smaller availability of space on the core particle leads to a lower probability of attaining the geometrically expected maximum valence in a given time interval.

## Conclusions

Reconfigurable colloidal molecules were assembled by combining two types of spherical colloids functionalized with complementary surface mobile DNA linkers in high number ratios. The size ratio of the constituent spheres determines the formation of flexible colloidal molecules with different geometrically determined number of bound particles, *N*_max_. The obtained *N*_max_ for each size ratio matched with the predicted maximum value for a closed packed configuration of hard spheres in 2D. The reconfigurability of the system thus solves the random parking problem that long hindered the formation of densely packed structures in earlier experiments. Additionally, the size ratio also determines the motion range of the colloidal molecules, which allowed us to tune the flexibility of the colloidal molecules. High yields of the geometrically predicted maximum valence of the core particle can be achieved for almost any size ratio and valence, although entropic effects make attaining close packed configurations nearly impossible. The growth dynamics of the colloidal molecules were examined in detail using experiments and agent-based simulations as well as analytic calculations. The assembly rate was observed to become slower as the colloidal molecules approached the maximum number of bound particles, owing to increasingly less available space on the core particles. For the same reason, smaller size ratios for a given maximum number of outer particles led to slower self-assembly. The experimental data showed good qualitative agreements with agent-based simulations and the analytical model. The high yields for any value of *N*_max_ and internal degrees of freedom make reconfigurable colloidal molecules an exciting model system for studying the behavior of flexible (bio)molecules such as intrinsically disordered proteins, immunoglobulins, or enzymes.^43−45^

The assembly strategy presented here might be extended to colloidal molecules with valence, that is, colloidal molecules whose outer particles possess a second type of interaction that enables interactions with other particles. We envision that this can be achieved by functionalizing the particles that form the outer lobes of the colloidal molecules with two DNA linkers with orthogonal interactions, one of which is being used to assemble the colloidal molecules and the other one of which is available for subsequent assembly of the colloidal molecule into a larger structure. After self-assembly of the colloidal molecules as described here, the secondary linkers would still be available for hierarchical binding, thereby imparting valence. Flexible colloidal molecules with and without valence have great potential to be utilized as the basic units for assembling complex, hierarchical structures, photonic crystals, and colloidal metamaterials and as model systems to study phase transitions by tuning the valences and the motion ranges.

## Methods

### Materials

Silica colloids (with diameters of 1.15 ± 0.05, 2.06 ± 0.05, 3.0 ± 0.25, and 7.0 ± 0.29 μm) were obtained commercially from Microparticles GmbH or synthesized in the laboratory by a modified Stöber’s method.^46^ The lipids 1,2 dioleoyl-*sn*-glycero-3-phosphocholine (DOPC), 1,2-dioleoyl-*sn*-glycero-3-phosphoethanolamine-*N*-[methoxy(polyethylene glycol)-2000] (ammonium salt) (DOPE-PEG_2000_), 1,2-dioleoyl-*sn*-glycero-3-phosphoethanolamine-*N*-(lissamine rhodamine B sulfonyl) (ammonium salt) (DOPE-rhodamine), and 1,2-dioleoyl-*sn*-glycero-3-phosphoethanolamine-*N*-(carboxyfluorescein) (ammonium salt) (DOPE-fluorescein) were obtained at >99% purity from Avanti Polar Lipids, Inc. Three different DNA strands (Eurogentec) with the following sequences were used (a) A strand: cholesterol-TEG-5′-TTT-ATC-GCT-ACC-CTT-CGC-ACA-GTC-AAT-CTA-GAG-AGC-CCT-GCC-TTA-CGA-*GTA-GAA-GTA-GG*-3′-6FAM; (b) B strand: cholesterol-TEG-5′-TTT-ATC-GCT-ACC-CTT-CGC-ACA-GTC-AAT-CTA-GAG-AGC-CCT-GCC-TTA-CGA-*CCT-ACT-TCT-AC*-3′-Cy3; (c) C strand: cholesterol-TEG-3′-TTT-TAG-CGA-TGG-GAA-GCG-TGT-CAG-TTA-GAT-CTC-TCG-GGA-CGG-AAT-GC-5′. The A and B strands consist of a backbone that can be hybridized with the C strand and an 11 base pair long sticky end with complementary sequences at the 3′ ends (denoted by italic characters). To identify the different linkers, A strands are labeled with the fluorescent dye 6-FAM, *i.e.*, 6-carboxyfluorescein (excitation: 488 nm; emission: 521 nm; depicted in green) and B is labeled with the fluorescent dye Cy3 (excitation: 561 nm; emission: 570 nm; depicted in magenta).

### Preparation of Silica Particles with Surface Mobile DNA Linkers

We functionalized silica particles with surface mobile DNA linkers using a protocol suggested by van der Meulen and Leunissen.^39^ At first, small unilamellar lipid vesicles (SUVs) were prepared from a mixture of DOPC and DOPE-PEG_2000_ in a 90:10 molar ratio in chloroform. If necessary, for increasing fluorescence, 0.001% mole fraction of DOPE-rhodamine or DOPE-fluorescein was added to label the membranes. The lipids were desiccated in vacuum and resuspended in HEPES buffer (10 mM HEPES, 47 mM NaCl, 3 mM NaN_3,_ pH = 7.01) for 30 min to obtain a 3 g/L solution. This was followed by 21 extrusions of the lipid mixture through two stacked polycarbonate filters (Whatman) with 30 nm pore size to obtain the SUVs. 50 μM solutions of the A or B DNA strand were hybridized with the C strand in a 1:1.5 volume ratio by heating the solution to 90 °C and cooling at 1 °C/min. The hybridized DNA strands consisted of a 47 base pair long double-stranded central part with double cholesterol anchors connected through TEG (tetraethylene glycol) spacers at one end and an 11 base pair long single-stranded sticky part at the other end, which could link to a complementary sticky end. The *S′* strand was produced by hybridizing the A and C strands, while *S* was produced by hybridizing the B and C strands.

At first, the SUVs were added to an equal volume of a 5 g/L solution of silica particles and put on a rotating tumbler for 40 min at a slow turn speed of 9 rotations/min in order to prevent sedimentation due to gravity. Then, the particles were centrifuged for 5 min at 494 rcf and washed with HEPES. Required amounts of DNA were added to the SUV encapsulated particles, and the resulting solution was again kept on the turner for 1 h. Finally, to get rid of any excess dye or lipids, the particles were washed three times in HEPES by centrifugation.

### Formation of Colloidal Molecules

To produce the colloidal molecules, two groups of particles with similar or different sizes and functionalized with complementary DNA were mixed together in a number ratio such that the designated core particles were surrounded by an excess of the other particles. We note that number ratios below 1:3 tend to produce a mixture of different geometries including colloidal molecules, colloidal polymers, and clusters without any definite configurations. At number ratios above 1:8 and above 1:20, mostly and only, respectively, colloidal molecules were obtained.

### Sample Observation

Flexible colloidal molecules were imaged in sample holders with a hydrophobized and passivated glass as the bottom surface. The glass surface was hydrophobized using Surfasil (a siliconizing agent) and passivated by adding 5% w/v Pluronic F-127 to the holder, storing it for 30 min, and rinsing with water. An inverted Nikon TI-E microscope equipped with a 100× objective lens (NA = 1.4) was used for both confocal and bright field imaging. Images were recorded using an A1R confocal scan head in 8 kHz resonant scan mode equipped with confocal GaAsp detectors and a monochrome CCD camera (DS-QiMc) in bright field mode. Excitation was achieved using a 40 mW argon laser (488 nm) and a 20 mW sapphire laser (561 nm).

### Simulations

Simulations following the algorithm outlined in the text were solved iteratively over time. To approach the maximum number of bound outer particles, 100 disks were placed in a periodic boundary at random locations (with no overlaps). 99 of these were selected to have radius *R*_0_, and the remaining ones had radius α*R*_0_; we set *R*_0_ = 1 and use this to define our length scale. The tethers were given length *R*_t_ = 0.02*R*_0_, which is consistent with typical length scales in experiments. The system size is rescaled to give a constant packing fraction of π/9 for all values of α. The size of steps in the random walk were *T* = 0.01 for all particles. The spring constants associated with collisions and stretching of the DNA tether, *K*_disk_ and *K*_tether_, respectively, were both set to 1, representing very stiff springs. The system was simulated for 10^6^ timesteps, and long simulations were necessary due to the low probability of reaching high valence. All simulation results presented here are the average of 500 independent realizations of the model.

## Supporting Information Available

Reconfigurable quasi-2D colloidal molecules (MP4)
Reconfigurable 3D colloidal molecules (MP4)
Colloidal molecules with different degrees of flexibility for *N*_max_ = 4 with size ratios (α) of 0.5 and 0.67 (MP4)
Agent-based simulation showing the growth of a colloidal molecule with α = 1 and saturation at a valence *N* = 5 (MP4)
Detailed analytic calculations along with schematic diagrams representing the analytical model (PDF)
