# Supplementary material for: Self-Assembly Dynamics of Reconfigurable Colloidal Molecules
Source: ACS Nano. 2022 Jan 26;16(2):2471–80. doi: 10.1021/acsnano.1c09088 (PMC8867909; doi:10.1021/acsnano.1c09088)
Supplement: Supplementary file 5 — nn1c09088_si_005.pdf [file nn1c09088_si_005.pdf]

Supporting Information

Self-assembly dynamics of reconfigurable colloidal molecules

Indrani Chakraborty[†,§], Daniel J. G. Pearce[‡,⁑,₴], Ruben W. Verweij[†], Sabine C. Matysik[†,§§], Luca Giomi[‡], and Daniela J. Kraft*[,†]

† Soft Matter Physics, Huygens-Kamerlingh Onnes Laboratory, Leiden Institute of Physics, PO Box 9504, 2300 RA Leiden, The Netherlands.

§ Department of Physics, Birla Institute of Technology and Science, Pilani - K K Birla Goa Campus, Zuarinagar, 403726, Goa, India

‡ Institute-Lorentz, Universiteit Leiden, PO Box 9506, 2300 RA Leiden, The Netherlands

⁑ Dept. of Mathematics, Massachusetts Institute of Technology, 182 Memorial Dr, Cambridge, Massachusetts, MA 02142, USA

₴ Dept. of Theoretical Physics, University of Geneva, Quai Ernest Ansermet 30, 1205 Geneva, Switzerland

§§ Yusuf Hamied Department of Chemistry, University of Cambridge, Lensfield Rd, Cambridge CB2 1EW, UK

* Corresponding Author: Email: Kraft@Physics.LeidenUniv.nl

Supporting Videos

Video S1: reconfigurable quasi-2D colloidal molecules

Video S2: reconfigurable 3D colloidal molecules

Video S3: video showing the different degrees of flexibility for two N_{max} = 4 colloidal molecules with size ratios α = 0.5 (left) and α = 0.67 (right).

Video S4: agent-based simulation showing the growth of a colloidal molecule with α = 1 and saturation at a valence N = 5.

Analytic Calculations

1 Average valence as function of time

We describe the adding of a colloidal particle to the central particle as a Poisson process. This implies that the average rate at which binding events occur is constant over time, though their actual addition is stochastic. The rate at which a cluster gains an Nth particle is constant and proportional to the rate at which the cluster interacts with a potential particle multiplied by the probability that the particle sticks. Since the number of free particles is significantly larger than the number of bound particles, we assume the rate at which the cluster meets other particles is constant and equal to 1/(time for particle to diffuse an average separation). However, the probability that an incoming particle is able to form a bond with the central particle depends on the current number of bound particles. This means the rate at which particles are added to the central cluster changes with the addition of each particle, we dub this process a sequential Poisson process.

Let t_0 be the average time between interactions between the cluster and a free particle and p_N be the probability that the incoming particle is able to form a bond with the cluster. Here p_N is a function of the current valence of the cluster, with p_N giving the probability of forming the Nth bond. Therefore, in a time interval of length Δt the expected number of particles to have interacted and bonded with a cluster of $N-1$ bound particles is $\lambda = \Delta t p_N / t_0$. Of course, in reality, once a particle has bonded, the value of p_N changes, thus the Poisson process starts again for the addition of the subsequent particle.

In order to derive an analytic description of the sequential Poisson process we start from the cumulative density function (CDF) of a standard Poisson process. This gives us the probability that there have been less than or equal to k events in a time interval in which we would expect λ events.

$$\boldsymbol{P(x \leq k) = e^{-\lambda} \sum_{i=0}^{\lfloor k \rfloor} \frac{\lambda^i}{i!}} \tag{1}$$

Therefore, the probability of no events occurring is $P(x \leq 0) = e^{-\lambda}$ and the probability that at least one event has happened is given by $P(x > 0) = 1 - e^{-\lambda}$. Therefore by substituting in for λ we define

$$C_N(\Delta t) = 1 - e^{-\Delta t p_N/t_0}. \tag{2}$$

This function is the CDF that a particle successfully binds to a cluster already containing $N - 1$ bound particles in a given time interval, Δt.

The generation of a single large cluster is the result of successive Poisson processes adding a single particle. Let us define the function $G_N(t)$ as the CDF of the Nth particle binding to a cluster which had zero bound particles at time $t = 0$. Therefore, the probability density function (PDF) of the same process is given by

$$F_N(t) = \partial_t G_N(t). \tag{3}$$

In order to calculate $G_N(t)$ we must consider the probability of adding an Nth particle given any possible time at which the $N - 1^{\text{th}}$ particle was added. This is generally given by

$$G_N(t) = \int_0^t C_N\,(t - t')F_{N-1}(t')dt'. \tag{4}$$

Since we know that the cluster has zero bound particles at time $t = 0$ we can trivially write

$$G_1(t) = 1 - e^{-tp_1/t_0}. \tag{5}$$

This can be used to inductively generate all subsequent functions G_N which are given by

$$G_2(t) = 1 + \frac{p_2 e^{-tp_1/t_0}}{(p_1 - p_2)} + \frac{p_1 e^{-tp_2/t_0}}{(p_2 - p_1)} \tag{6}$$

$$G_3(t) = 1 - \frac{p_2 p_3 e^{-tp_1/t_0}}{(p_1 - p_2)(p_1 - p_3)} - \frac{p_1 p_3 e^{-tp_2/t_0}}{(p_2 - p_1)(p_2 - p_3)} - \frac{p_1 p_2 e^{-tp_3/t_0}}{(p_3 - p_1)(p_3 - p_2)} \tag{7}$$

$$G_4(t) = 1 + \frac{p_2 p_3 p_4 e^{-tp_1/t_0}}{(p_1 - p_2)(p_1 - p_3)(p_1 - p_4)} + \frac{p_1 p_3 p_4 e^{-tp_2/t_0}}{(p_2 - p_1)(p_2 - p_3)(p_2 - p_4)} + \frac{p_1 p_2 p_4 e^{-tp_3/t_0}}{(p_3 - p_1)(p_3 - p_2)(p_3 - p_4)} + \frac{p_1 p_2 p_3 e^{-tp_4/t_0}}{(p_4 - p_1)(p_4 - p_2)(p_4 - p_3)} \tag{8}$$

$$G_5(t) = 1 - \frac{p_2 p_3 p_4 p_5 e^{-tp_1/t_0}}{(p_1 - p_2)(p_1 - p_3)(p_1 - p_4)(p_1 - p_5)} - \frac{p_1 p_3 p_4 p_5 e^{-tp_2/t_0}}{(p_2 - p_1)(p_2 - p_3)(p_2 - p_4)(p_2 - p_5)} - \frac{p_1 p_2 p_4 p_5 e^{-tp_3/t_0}}{(p_3 - p_1)(p_3 - p_2)(p_3 - p_4)(p_3 - p_5)} - \frac{p_1 p_2 p_3 p_5 e^{-tp_4/t_0}}{(p_4 - p_1)(p_4 - p_2)(p_4 - p_3)(p_4 - p_5)} - \frac{p_1 p_2 p_3 p_4 e^{-tp_5/t_0}}{(p_5 - p_1)(p_5 - p_2)(p_5 - p_3)(p_5 - p_4)}. \tag{9}$$

This can be generalized for larger N as

$$G_N(t) = 1 + (-1)^N \sum_{i=1}^{N} Q_i^N \, e^{-tp_i/t_0} \tag{10}$$

where the coefficients are given by

$$Q_i^N = \begin{cases} 1, & \text{for } N = 1 \\ \frac{\prod_{j \neq i}^{n} p_j}{\prod_{j \neq i}^{n} (p_i - p_j)}, & \text{for } N \neq 1. \end{cases} \tag{11}$$

The expected valence as a function of time can then be written as the sum of the CDFs, since each Poisson process increases the valence by 1, and hence

$$\langle V(t) \rangle = \sum_{i=1}^{N_{\max}} G_N(t) \tag{12}$$

where $N_{\max}$ is the maximum valence allowed given the relative size of the inner and outer particles. By definition $G_N(t) = 0$ for $N > N_{\max}$. The prediction of Eq.12 is plotted alongside a simulated sequential Poisson process in Figure S1.

1.1 Probability density function of residence time at each valence

The PDF of the time spent at each valence can be written as

$$\boldsymbol{R_N(\Delta t) = \frac{p_N}{(p_N - p_{N+1})}\left[e^{-\Delta t p_{N+1}/t_0} - e^{-\Delta t p_N/t_0}\right]}. \tag{13}$$

This can be derived by recognizing that $R_1 = G_1 - G_2$, which can be generalized for any N by substituting in for p_N.

1.2 Probability of cluster having a certain valence

This is simply the difference between two subsequent CDFs.

$$\boldsymbol{P(N, t) = G_N(t) - G_{N+1}(t)}. \tag{14}$$

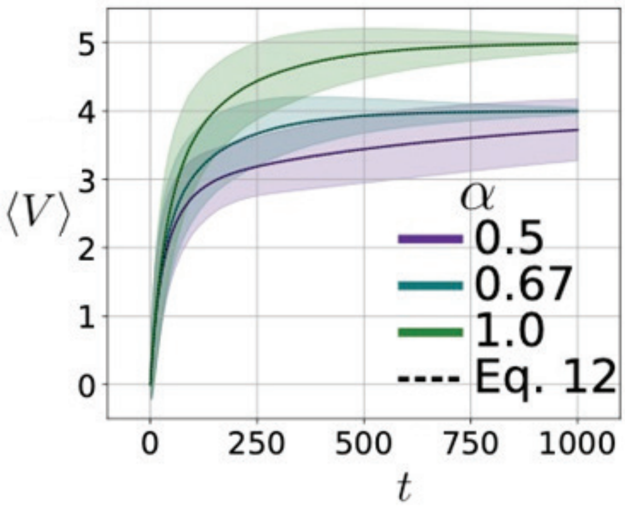

Figure S1. Equation 12 plotted along a simulated sequential Poisson process using the values of $\boldsymbol{p_N}$ given in Table I below.

2. Probability of Nth particle sticking

The probability of each particle sticking is given in the tables below. These are calculated using a statistical approach considering the average available angle for an incoming particle to bind. The first table (Table I) gives the results in 2D in which sedimentation is not considered whereas the second table gives the results when particle sedimentation is considered.

Table I. Probability of Nth particle sticking for α = 1.0 , α = 0.67 and α = 0.5 without considering sedimentation

N	α = 1.0	α = 0.67	α = 0.5
1	1	1	1
2	0.670	0.594	0.543
3	0.381	0.258	0.182
4	0.179	0.062	0.014
5	0.047	-	-

Table II. Probability of Nth particle sticking for α = 1.0 , α = 0.67 and α = 0.5 considering sedimentation

N	α = 1.0	α = 0.67	α = 0.5
1	1	1	1
2	0.670	0.584	0.509
3	0.381	0.242	0.137
4	0.179	0.050	0.001
5	0.047	-	-

2.1 Calculating the binding probabilities

We take a simple probabilistic approach to recreate the valence curves generated by simulations and experiments. We assume that an incoming particle arrives from a random angle and binds with some probability related to the available space given the number of particles already bound.

The problem is set up as follows, the central particle has radius αR_0 and is at fixed position (0,0). The bound particles have radius R_0 and their angular positions are given by θ. The bound particles are numbered and have angular positions θ_i and without loss of generality we set $\theta_1 = -\varphi$ where φ is the minimal angle between two bound particles, e.g. $\varphi = \pi/3$ for hexagonal close packed spheres; this sets the origin, see Figure S2a-b.

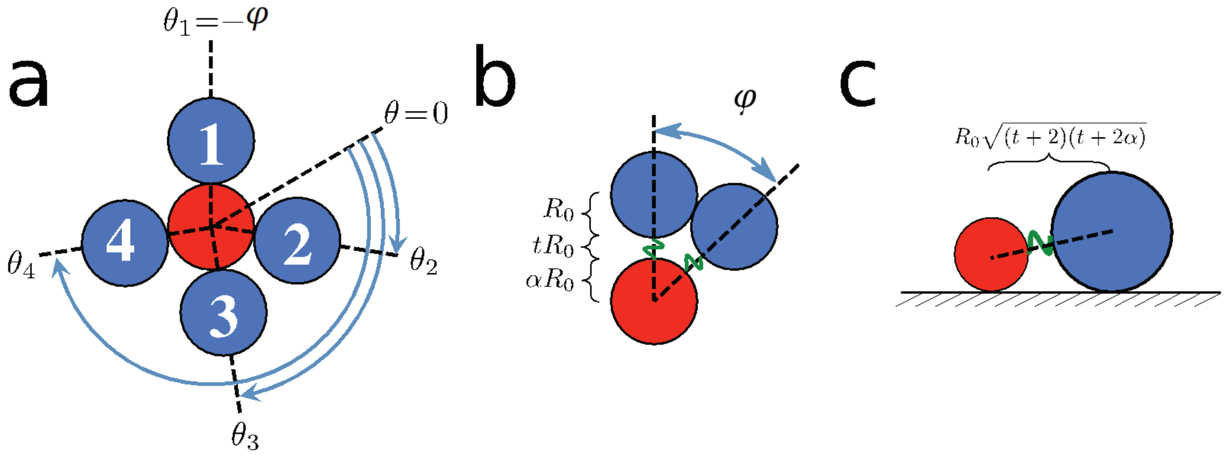

Figure S2. (a) Diagram of the variables used in the calculation. (b) Diagram of the definition of φ. (c) Diagram detailing how the definition of φ changes due to the 3D sedimentation of the particles.

2.2 Defining the minimum angle φ between two bound particles

We define the angle φ as the minimum angle between two bound particles. It is essential here to include the tether length in the calculation of φ, which is defined as $2R_t = t \times R_0$. This is defined by simply applying the cosine rule to the arrangement given in Figure S2b. We also make the assumption that $t << min[1, \alpha]$ in which case the minimum angle is achieved when both tethers are fully extended.

This gives the angle as

$$\cos(\boldsymbol{\varphi}) = \mathbf{1} - \mathbf{2}(\boldsymbol{\alpha} + \boldsymbol{t} + \mathbf{1})^{-\mathbf{2}}. \tag{15}$$

By using the identity $cos(\varphi) = 1 - 2sin^2\frac{\varphi}{2}$, we can rewrite this as:

$$\mathbf{sin}\left(\frac{\boldsymbol{\varphi}}{\mathbf{2}}\right) = \frac{\mathbf{1}}{\mathbf{1} + \boldsymbol{\alpha} + \boldsymbol{t}}. \tag{16}$$

If we make the assumption that the centers of all the particles are at the same height, the maximum number of outer particles that can fit around the central particle is given by $N_{max} = \frac{2\pi}{\varphi}$. Substituting in for the above expression gives:

$$\boldsymbol{N_{max}} = \frac{\boldsymbol{\pi}}{\mathbf{arcsin}\left(\frac{\mathbf{1}}{\mathbf{1}+\boldsymbol{\alpha}+\boldsymbol{t}}\right)}. \tag{17}$$

For close-packed hard spheres, the tether length is zero ($t = 0$) and we arrive at Eq. (1) from the main text.

While this estimate of φ is good for the simulations, which take place in a strictly 2D environment, for the experiments it must be updated to account for the change in height of the centers of the spheres as they sediment onto the surface, see Figure S2c. Now the maximum in plane distance between the centers of two spheres is given by $R_0\sqrt{(t+2)(t+2\alpha)}$ and the minimum angle between joined spheres is again given by:

$$\cos(\boldsymbol{\varphi}) = \mathbf{1} - \mathbf{2}\left((\boldsymbol{t}+\mathbf{2})(\boldsymbol{t}+\mathbf{2}\boldsymbol{\alpha})\right)^{-\mathbf{1}}. \tag{18}$$

Following a similar argument to above, this leads to an updated maximum number of neighbors around a central sphere when resting on a flat surface.

$$\boldsymbol{N}_{\mathbf{max}}^{\boldsymbol{S}} = \frac{\boldsymbol{\pi}}{\mathbf{arcsin}\left(\frac{\mathbf{1}}{\sqrt{(\boldsymbol{t}+\mathbf{2})(\boldsymbol{t}+\mathbf{2}\boldsymbol{\alpha})}}\right)}. \tag{19}$$

2.3 Finding the binding probabilities

The probability of p_1 binding is 100% since all angles are unobstructed in all configurations of the system. Therefore $P(p_1) = 1$. This of course gets more complicated as more particles are added to the system.

2.3.1 Adding particle 2

For particle 2, more care is needed. Since particle 1 is already bound, it will be in the way of some trajectories to the central particle. A single particle occludes an arc of 2φ as the minimum angular

separation of two bound particle is φ, hence the available angular region is $2\pi - 2\varphi$. If we normalise this by the total angle that particle 2 could approach from, we get $P(p_2) = \frac{2\pi-2\varphi}{2\pi}$. In reality we should integrate over all possible configurations of p_1, so we actually get:

$$\boldsymbol{P(p_2) = \frac{1}{2\pi}\int_0^{2\pi} \frac{2\pi - 2\varphi}{2\pi} d\theta_1 = \frac{2\pi - 2\varphi}{2\pi}}. \tag{20}$$

2.3.2 Adding particle 3

For the third particle the available space depends on the configuration of the previous two bound particles. We will choose a regime here in which we scan through all configurations by moving the particles one by one from their minimal angular position to their maximal position allowed by the other bound particles. Without any loss of generality, we assume that the initial positions of the particles are given by $\theta'_i = (i-2) \times \varphi$. We scan through all possible configurations of the system by scanning through the maximal allowed range of θ for each particle aside from $\theta_1 = -\varphi$ which defines our origin. Scanning through θ_1 which would correspond to rotating our whole system therefore has no effect on available space. This is analogous to scanning through all possible configurations of an abacus. This is an arbitrary choice but makes the problem easier to visualize and the mathematics simpler to formulate.

We now split the problem into two cases which correspond to the potential gaps between the particles. These are outlined below in the schematic in Figure S3.

Case 1: $\theta_2 \in [0, 2\pi - 3\varphi]$ (Fig. S3a):

- Probability of p_2 being in this region = $(2\pi - 3\varphi)/(2\pi - 2\varphi)$
- Consider the space between particle 2 and 1 in the region $\theta \in [\theta_2 + \varphi, 2\pi - 2\varphi]$.
- Available space for an incoming particle = $2\pi - 3\varphi - \theta_2$

Therefore, this gives a probability of $\frac{2\pi-3\varphi}{2\pi-2\varphi}\frac{1}{\int_0^{2\pi-3\varphi} d\theta_2}\int_0^{2\pi-3\varphi}\frac{1}{2\pi}(2\pi - 3\varphi - \theta_2)d\theta_2$

Case 2: $\theta_2 \in [\varphi, 2\pi - 2\varphi]$ (Fig. S3b):

- Probability of p_2 being in this region = $(2\pi - 3\varphi)/(2\pi - 2\varphi)$
- This is in fact the mirror of Case 1, with space in regions $\theta \in [0, \theta_2 - \varphi]$.
- Available space for an incoming particle = $\theta_2 - \varphi$

Therefore, this gives a probability of $\frac{2\pi-3\varphi}{2\pi-2\varphi}\frac{1}{\int_{\varphi}^{2\pi-2\varphi}d\theta_2}\int_{\varphi}^{2\pi-2\varphi}\frac{1}{2\pi}(\theta_2-\varphi)d\theta_2$

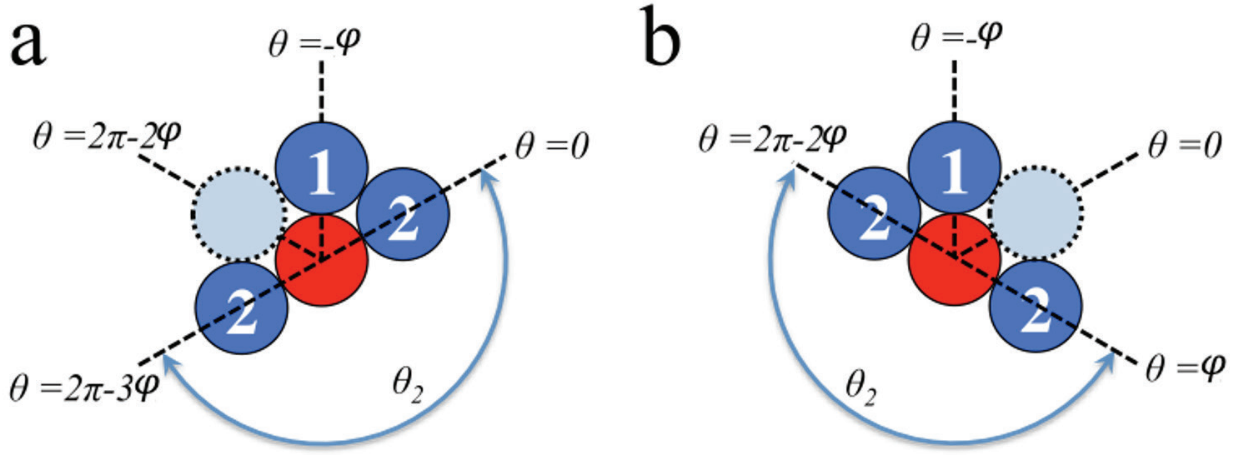

Figure S3. Diagram showing the range of θ_2 in which case 1 and case 2 applies. Space for a new particle in regions (a) $\theta \in [\theta_2 + \varphi, 2\pi - 2\varphi]$ and (b) $\theta \in [0, \theta_2 - \varphi]$.

The normalization for the integrations will cancel with the available arc for p_2 in all cases and we get:

$$\boldsymbol{P(p_3) = \frac{1}{2\pi(2\pi-2\varphi)}\left[\int_0^{2\pi-3\varphi}(2\pi-3\varphi-\theta_2)\,d\theta_2 + \int_{\varphi}^{2\pi-2\varphi}(\theta_2-\varphi)\,d\theta_2\right]}. \quad (21)$$

This can be solved to give:

$$\boldsymbol{P(p_3) = \frac{(3\varphi-2\pi)^2}{2\pi(2\pi-2\varphi)}}. \quad (22)$$

2.3.3 Adding particle 4

Now we must consider the relative positions of three particles, so we have two independent variables and three possible spaces.

Case 1: $\theta_3 \in [\varphi, 2\pi-3\varphi]$:

- Probability of p_3 being in this region = $(2\pi-4\varphi)/(2\pi-3\varphi)$
- Consider space between particle 1 and 3 in the region $\theta \in [\theta_3+\varphi, 2\pi-2\varphi]$.
- Available space for an incoming particle = $2\pi-3\varphi-\theta_3$.

Case 2: $\theta_3 \in [2\varphi, 2\pi-2\varphi]$ and $\theta_2 \in [0, \theta_3-2\varphi]$.

- Probability of p_3 being in this region = $(2\pi - 4\varphi)/(2\pi - 3\varphi)$
- Probability of p_2 being in this region = $\frac{\theta_3 - 2\varphi}{\theta_3 - \varphi}$.
- Consider space between particle 2 and 3 in the region $\theta \in [\theta_2 + \varphi, \theta_3 - \varphi]$.
- Available space for an incoming particle = $\theta_3 - 2\varphi - \theta_2$.

Case 3: $\theta_3 \in [2\varphi, 2\pi - 2\varphi]$ and $\theta_2 \in [\varphi, \theta_3 - \varphi]$:

- Probability of p_3 being in this region = $(2\pi - 4\varphi)/(2\pi - 3\varphi)$
- Probability of p_2 being in this region = $\frac{\theta_3 - 2\varphi}{\theta_3 - \varphi}$.
- Consider space between particle 1 and 2 in the region $\theta \in [0, \theta_2 - \varphi]$.
- Available space for an incoming particle = $\theta_2 - \varphi$.

The probabilities can be constructed similarly to above to obtain:

$$\boldsymbol{P(p_4) = \frac{1}{2\pi(2\pi - 3\varphi)}\left[\int_{\varphi}^{2\pi - 3\varphi} d\theta_3 (2\pi - 3\varphi - \theta_3) + \int_{2\varphi}^{2\pi - 2\varphi} d\theta_3 \left(\frac{1}{\theta_3 - \varphi}\int_0^{\theta_3 - 2\varphi} d\theta_2 (\theta_3 - 2\varphi - \theta_2)\right) + \int_{2\varphi}^{2\pi - 2\varphi} d\theta_3 \left(\frac{1}{\theta_3 - \varphi}\int_{\varphi}^{\theta_3 - \varphi} d\theta_2 (\theta_2 - \varphi)\right)\right]} \tag{23}$$

which can be solved to give:

$$\boldsymbol{P(p_4) = \frac{4\pi^2 - 18\pi\varphi + 20\varphi^2 + \varphi^2 log\left[\frac{2\pi - 3\varphi}{\varphi}\right]}{2\pi(2\pi - 3\varphi)}}. \tag{24}$$

2.3.4 Adding particle 5

This process can be extended to arbitrary many particles added to the system, although with each new particle another layer of integration is added and the problem becomes more complex. We will stop at p_5 since we are looking primarily at size ratios $\alpha \leq 1$, so the maximum valence we expect to obtain is 6 for hexagonal close packing.

There are now 3 independent variables which gives rise to four cases.

Case 1: $\theta_4 \in [2\varphi, 2\pi - 3\varphi]$

- Consider space between particle 1 and 4 in the region $\theta \in [\theta_4 + \varphi, 2\pi - 2\varphi]$.
- Probability of p_4 being in this region = $(2\pi - 5\varphi)/(2\pi - 4\varphi)$
- Available space for an incoming particle = $2\pi - 3\varphi - \theta_4$.

Case 2: $\theta_4 \in [3\varphi, 2\pi - 2\varphi]$ and $\theta_3 \in [\varphi, \theta_4 - 2\varphi]$

- Probability of p_4 being in this region = $(2\pi - 5\varphi)/(2\pi - 4\varphi)$
- Probability of p_3 being in this region = $(\theta_4 - 3\varphi)/(\theta_4 - 2\varphi)$ (given θ_4)
- Consider space between particle 3 and 4 in the region $\theta \in [\theta_3 + \varphi, \theta_4 - \varphi]$.
- Available space for an incoming particle = $\theta_4 - 2\varphi - \theta_3$.

Case 3: $\theta_4 \in [3\varphi, 2\pi - 2\varphi]$ and $\theta_3 \in [2\varphi, \theta_4 - \varphi]$ and $\theta_2 \in [0, \theta_3 - 2\varphi]$

- Probability of p_4 being in this region = $(2\pi - 5\varphi)/(2\pi - 4\varphi)$
- Probability of p_3 being in this region = $(\theta_4 - 3\varphi)/(\theta_4 - 2\varphi)$ (given θ_4)
- Probability of p_2 being in this region = $(\theta_3 - 2\varphi)/(\theta_3 - \varphi)$ (given θ_3)
- Consider space between particle 2 and 3 in the region $\theta \in [\theta_2 + \varphi, \theta_3 - \varphi]$.
- Available space for an incoming particle = $\theta_3 - 2\varphi - \theta_2$.

Case 4: $\theta_4 \in [3\varphi, 2\pi - 2\varphi]$ and $\theta_3 \in [2\varphi, \theta_4 - \varphi]$ and $\theta_2 \in [\varphi, \theta_3 - \varphi]$

- Probability of p_4 being in this region = $(2\pi - 5\varphi)/(2\pi - 4\varphi)$
- Probability of p_3 being in this region = $(\theta_4 - 3\varphi)/(\theta_4 - 2\varphi)$ (given θ_4)
- Probability of p_2 being in this region = $(\theta_3 - 2\varphi)/(\theta_3 - \varphi)$ (given θ_3)
- Consider space between particle 1 and 2 in the region $\theta \in [0, \theta_2 - \varphi]$.
- Available space for an incoming particle = $\theta_2 - \varphi$.

This can be combined to give the formula:

$$P(p_5) = \frac{1}{2\pi(2\pi - 4\varphi)}\Bigg[\int_{2\varphi}^{2\pi-3\varphi} d\,\theta_4(2\pi - 3\varphi - \theta_4) + \int_{3\varphi}^{2\pi-2\varphi} d\,\theta_4\left(\frac{1}{\theta_4 - 2\varphi}\int_{\varphi}^{\theta_4-2\varphi} d\,\theta_3(\theta_4 - 2\varphi - \theta_3)d\theta_3\right)d\theta_4 + \int_{3\varphi}^{2\pi-2\varphi} d\,\theta_4\left(\frac{1}{\theta_4 - 2\varphi}\int_{2\varphi}^{\theta_4-\varphi} d\,\theta_3\left(\frac{1}{\theta_3 - \varphi}\int_{0}^{\theta_3-2\varphi} d\,\theta_2(\theta_3 - 2\varphi - \theta_2)\right)\right) + \int_{3\varphi}^{2\pi-2\varphi} d\,\theta_4\left(\frac{1}{\theta_4 - 2\varphi}\int_{2\varphi}^{\theta_4-\varphi} d\,\theta_3\left(\frac{1}{\theta_3 - \varphi}\int_{\varphi}^{\theta_3-\varphi} d\,\theta_2(\theta_2 - \varphi)\right)\right)\Bigg] \tag{25}$$

which can be solved to give:

$$P(p_5) = \frac{4\pi^2 - 24\varphi\pi + 35\varphi^2 + 2\varphi^2\log\left[\frac{2\pi - 4\varphi}{\varphi}\right] + \frac{\varphi^2}{4}\left(\log\left[\frac{2\pi}{\varphi} - 4\right]\right)^2}{2\pi(2\pi - 4\varphi)}. \tag{26}$$

We can now substitute in φ for the different cases we are looking at and find the probabilities given in the table earlier.

2.4 Generating function for arbitrary valence

It is possible to write a generating function for the probability of addition of particles to a system with arbitrary currently bound particles. First, we need to introduce some new notation. We define $\theta_i{}'$ as the initial position of particle i, which under our current set up is $\theta_i{}' = (i-2)\varphi$. For a system with N currently bound particles, we only use $N-1$ free parameters as $\theta_1 = -\varphi$. In order to accommodate the periodicity in the system we must define $\theta_{N+1} = 2\pi - \varphi$ and the initial positions of these as $\theta_1{}' = -2\varphi$ and $\theta_{N+1}{}' = (N-1)\varphi$.

This equation will obviously only hold when the minimum angle between adjacent particles is small enough to accommodate all the particles currently bound, i.e. $(N+1)\varphi < 2\pi$, otherwise it is impossible to add an additional particle.

The generating function for the probability of adding particle $N+1$ is then:

$$\boldsymbol{P(p_{N+1}) = \frac{1}{2\pi}\sum_{i=1}^{N}\left[\overleftarrow{\prod_{j=i+1}^{N+1}} g_j \circ\right]\frac{1}{\theta_{i+1}-\theta_{i+1}{}'}\int_{\theta_i{}'}^{\theta_{i+1}-2\varphi}(\theta_{i+1}-2\varphi-\theta_i)\,d\theta_i} \tag{27}$$

where g_j is a function defined as follows:

$$\boldsymbol{g_j(f)} = \begin{cases} \boldsymbol{f}, & \text{if } \boldsymbol{j = N+1} \\ \boldsymbol{\frac{1}{\theta_{j+1}-\theta_{j+1}{}'}\int_{\theta_j{}'+\varphi}^{\theta_{j+1}-\varphi} f\,d\theta_j}, & \text{otherwise} \end{cases} \tag{28}$$

and f_i is a function defined as

$$\boldsymbol{f_i = \frac{1}{\theta_{i+1}-\theta_{i+1}{}'}\int_{\theta_i{}'}^{\theta_{i+1}-2\varphi}(\theta_{i+1}-2\varphi-\theta_i)\,d\theta_i} \tag{29}$$

where the order of the product in Eq. 27 is indicated by the arrow, hence the product is a function which acts on the final integral

$$\begin{aligned}
&\boldsymbol{\left[\overleftarrow{\prod_{j=i+1}^{N+1}} g_j \circ\right]\frac{1}{\theta_3-\theta_3{}'}\int_{\theta_2{}'}^{\theta_3-2\varphi}(\theta_3-2\varphi-\theta_2)\,d\theta_2} \\
&\boldsymbol{= g_5\left(g_4\left(g_3\left(\frac{1}{\theta_3-\theta_3{}'}\int_{\theta_2{}'}^{\theta_3-2\varphi}(\theta_3-2\varphi-\theta_2)\,d\theta_2\right)\right)\right)} \\
&\boldsymbol{= \frac{1}{\theta_6-\theta_6{}'}\int_{\theta_5{}'+\varphi}^{\theta_6-\varphi}\frac{1}{\theta_5-\theta_5{}'}\int_{\theta_4{}'+\varphi}^{\theta_5-\varphi}\frac{1}{\theta_4-\theta_4{}'}\int_{\theta_3{}'+\varphi}^{\theta_4-\varphi}\frac{1}{\theta_3-\theta_3{}'}\int_{\theta_2{}'}^{\theta_3-2\varphi}(\theta_3-2\varphi-\theta_2)\,d\theta_2 d\theta_3 d\theta_4 d\theta_5}.
\end{aligned} \tag{30}$$
